# Low Selenium Levels in Amniotic Fluid Correlate with Small-For-Gestational Age Newborns

**DOI:** 10.3390/nu12103046

**Published:** 2020-10-05

**Authors:** Ksenija Ogrizek-Pelkič, Monika Sobočan, Iztok Takač

**Affiliations:** 1 Faculty of Medicine, Department of Obstetrics and Gynaecology, University of Maribor, Taborska ulica 8, 2000 Maribor, Slovenia; ksenija.pelkic@gmail.com (K.O.-P.); iztok.takac@ukc-mb.si (I.T.); 2 University Medical Centre Maribor, Division of Gynaecology and Perinatology, Ljubljanska ulica 5, 2000 Maribor, Slovenia

**Keywords:** selenium, amniotic fluid, small-for-gestational-age newborns

## Abstract

Background: Identifying women at risk for small-for-gestational-age newborns (SGA) is an important challenge in obstetrics. Several different risk factors have been suggested to contribute to the development of SGA. Previous research is inconclusive on the role selenium (Se) plays in the development of SGA. The aim of the study was therefore to explore the role of Se concentrations in amniotic fluid in order to understand its possible role in the development of SGA. Study Design: This prospective, single center study investigated the relationships between Se concentrations in amniotic fluid and pregnancy outcomes. Amniotic fluid was collected from pregnant women during amniocentesis at 16/17 weeks of pregnancy. Se values were determined using the electrothermal atomic absorption spectrometry and expressed in µg/L. Characteristics of mothers and newborns were obtained from women and delivery records. Results: 327 samples of amniotic fluid were evaluated. Patients with SGA newborns had significantly lower mean values of amniotic fluid concentrations of Se compared to appropriate-for-gestational-age (AGA) newborns (4.8 ± 1.9 µg/L versus 5.6 ± 2.5 µg/L (*p* = 0.017)). Adjusting for different risk factors, Se remained the only significant factor impacting the outcome of a newborn (b = −0.152, s.e. = 0.077; *p* < 0.048). Se levels in amniotic fluid did not correlate with pre-eclampsia or preterm delivery. Conclusion: Amniotic fluid Se levels represent a viable root of further investigation and assessment in order to identify women with low birth weight newborns early. Women with decreased Se levels had a statistically significant chance of developing SGA. Further research is needed to elucidate the link between Se, other trace elements, and other risk factors and their impact on the development of SGA newborns.

## 1. Introduction

Small-for-gestational-age (SGA) newborns are defined as babies below the 10th percentile of an individualized birth-weight ratio. Newborns with SGA are associated with increased perinatal and neonatal morbidity and mortality [1,2]. The incidence of SGA is estimated to be approximately 5–10% in the general obstetrics population [2,3]. In SGA newborns, the most significant problem is perinatal asphyxia involving multiple organ systems [4]. The permanent changes in structure and metabolism due to SGA have also been attributed to the increased risk of adult chronic disease (cardiovascular diseases, type 2 diabetes) [4]. However, it is difficult to identify women who are early in pregnancy in which fetuses with SGA will develop [5]. An important risk factor for SGA as well as pre-eclampsia and pre-term birth, adjusted for other confounding factors, is advanced maternal age [6]. Therefore, especially in this group of pregnant women improved indicators for identifying fetuses at risk for SGA are needed.

One root of investigation is that of assessing trace element levels and their impact on the fetus. Trace elements have the role to enable proper activity of biochemical and enzymatic reactions [7]. The appropriate supply of trace elements in pregnancy is hypothesized to be crucial for a healthy pregnancy [8]. Previous research shows, that not only the appropriate levels of folate and vitamin D, which were extensively studied, but also trace minerals, such as iron, zinc, copper, and others, are needed to support a pregnancy [9]. Deficiency in copper and zinc were shown to be connected to teratogenic effects through the mechanism of enzyme disfunction, as key enzymes require these metals to function [9].

Changes in fetal development are reflected in disorders of the placenta (morphological and functional) [7]. Studies regarding the tissue-specific selenoprotein expression show that especially the iodotyronine deiodinase (DIOs) expression was expressed in developing maternal tissues such as the placenta as well as the fetus. The expression of DIO3 was shown through animal models to critically impact fetal thyroid hormone and regulate growth [10]. In early pregnancy, studies further on show, that selenium has positive effects on trophoblast cells through improving their viability and migration especially if exposed to hypoxia. In selenium-treated cells, mitochondrial membrane potential was increased, and reactive oxygen species levels decreased [11].

Selenium (Se) has been identified as one of the essential trace elements most connected to human health [12]. Se is an essential trace element for normal growth and development [13]. Se-containing enzymes can contribute to the attenuation of excessive inflammation [12]. During pregnancy, maternal Se concentrations and glutathione peroxidase (GPx) activity decrease [14,15,16,17]. Research shows, Se as well as cadmium (Cd) antagonistically work against oxidative stress by impacting redox balance and apoptotic signaling [18]. Deficiencies in Se were also linked to different diseases such as diabetes, heart disease, autoimmune disease, and even cancer. This might be based on the impact Se has on genes related to insulin, inflammation, and lipid metabolism [12].

Selenium deficiency has been reported in common chronic diseases such as hypothyroidism [19].

Several disorders during pregnancy have been linked to Se deficiency. These disorders include pre-eclampsia, gestational diabetes mellitus (GDM), neural tube defects, fetal growth restriction, and preterm birth [20].

Pregnancy represents a physiological state, which is considered to be a time of increased oxidative stress and in order to protect the fetus, the maternal antioxidants need to work against free radicals in utero [21]. During pregnancy, especially pre-eclamptic women, who often exhibit SGA newborns, have reduced placental GPx activity compared to control women [17]. There is with high likelihood a link between low selenium (Se) and pre-eclampsia. Women who had reduced levels of Se in umbilical venous blood compared to normal pregnancy had a significantly higher amount of pre-eclampsia [17], thus presenting a viable root of discovering fetuses that could possibly develop SGA [22]. Several lines of evidence support the oxidative hypothesis in the pathogenesis of pre-eclampsia, with placental underperfusion leading to an excessive production of reactive oxygen species (ROS) in lipid peroxides causing oxidative stress and endothelial cell dysfunction [23,24,25,26,27,28]. Lower levels of Se were observed in the status of pre-eclamptic mothers when compared to normal controls [29]. Low plasma Se concentration could contribute to the risk of delivering an SGA newborn, possibly through lowering placental antioxidant defense, thus directly affecting fetal growth [14,24]. It has been suggested that reduced Se concentration results in reduced GPx activity culminating in reduced antioxidant protection of biological membranes and DNA during the early stages of embryonic development [30].

The impact of Se is, however, not only hypothesized to be on the development of pre-eclampsia, but also on premature birth, intrauterine growth restrictions, and gestational diabetes [22]. Everson et al. performed a study evaluating the role of placental heavy metals (cadmium (Cd), arsenic (As), mercury (Hg), and lead (Pb)) as well as the essential trace elements zinc (Zn) and Se on the placenta. They showed that for placentas with low Zn and Se values, the weight of the placenta was also decreased [18,31]. Lewandowska et al. showed that determining levels of Se after adjustment for other trace elements in early pregnancy (10th–14th week of gestation) still predicted well SGA newborns, which had reduced levels of Se in comparison with normal Se levels. They, however, also pointed out that pre-pregnancy BMI levels importantly affected SGA [5].

Recent evidence points towards the presence of selenium-low or selenium-high diets during pregnancy and lactation being connected to the development of insulin resistance in pups. A selenium-high diet led to high insulin secretion, obesity, inflammation, and low leptin levels, showing that intrauterine Se levels might impact life far beyond the first weeks after birth [32]. The same research group then showed that when comparing rats with low- and high-selenium diets, there are deep changes in the adenosine monophosphate (AMP)-activated protein kinase (AMPK), changed homeostasis of Se, selenoproteins, IRS-1 expression, insulin levels, and oxidative balance which might lead to insulin resistance [33]. The same research group determined even further that when exposed to a fructose-high environment leading to a metabolic syndrome in rats, there were low levels of Se in their circulation which lead to processes similar to type 1 diabetes, indicating that Se might be an important marker for such disorders during gestation [34].

There have been different attempts to evaluate the levels of Se and their impact on the development of SGA newborns. Next to serum Se levels [5], an important root of investigation is also the impact of Se present in amniotic fluid (AF). AF is swallowed by the fetus and thus provides one of the most important roots of fetus nutrients impacting fetal absorption and growth [35]. Most studies evaluating Se levels are done by evaluating serum levels of Se; however, Yildrim et al. showed that there were significantly different concentrations of Se in maternal blood, fetal blood from the umbilical vein, maternal urine, and amniotic fluid. It was shown, that maternal blood and fetal blood correlate as well as urine levels and amniotic fluid levels [36]. However, for understanding the specific effects of Se on mesenchymal stem cells (MSCs) present in the amniotic fluid, the exploration of amniotic levels of Se was important to further understand this microelements relationship to growth. Studies show that ROS impacts cellular senescence and that this process is mediated by antioxidants such as glutathione, ascorbic acid, and ROS connected enzymes. Thus, by reducing ROS levels, one could improve DNA damage, prevent apoptosis, and recover mitochondrial dysfunction [37]. Therefore, in order to better understand the biological background Se has on growth amniotic fluid, it is an important biological material to study.

Our study aimed to investigate the impact of Se concentration in AF of pregnant women referred to amniocentesis due to an increased risk of adverse pregnancy outcomes. The primary outcome of the study was to assess the risk low Se levels bear for adverse outcomes. Through evaluating our primary aim, we examined the possibility to use amniotic fluid levels of microelements such as Se in order to improve our risk assessment for women at risk of their fetuses developing towards SGA.

## 2. Materials and Methods

### 2.1. Patient Selection

Women being monitored during pregnancy at the Department of Perinatology, University Medical Centre Maribor (UMC Maribor) were invited to participate in this study. We invited volunteers which had been referred to an amniocentesis at 16/17 weeks of pregnancy in accordance to national guidelines for prenatal diagnostics. The patients were referred to an amniocentesis based on the rules for carrying out preventive health care at the primary level issued by the government of Slovenia either due to the advanced age (amniocentesis is recommended for women above 37 years of age), due to increased risk for anomalies based on non-invasive testing methods, or due to any clinical suspicion (e.g., abnormal ultrasound) that warranted amniocentesis. If an anomaly was confirmed by the amniocentesis, the woman was excluded from further analysis in this study. All women in our study had singleton pregnancies. Women who received multivitamins containing Se were also excluded from the study.

The diagnosis of SGA was confirmed postnatally, based on the criteria of fetal birth weight below the 10th centile of a growth curve adjusted for the Slovenian population. To increase the precision during the selection of those cases with SGA newborns, the authors of this study used the birth weight criterion <10th on the Verdenik Curve [38] and associated it to a second nutritional classification criterion, namely the BWR ratio < 0.85. This means newborns who were small but nevertheless normal were excluded [39]. All newborns were assessed using the Verdenik Curve.

### 2.2. Data Analysis and Biological Sample Processing

Patient demographic data as well as amniotic fluid during ultrasound guided amniocentesis was collected and analyzed between the years 2011 and 2015. We collected 18–20 mL of amniotic fluid which was centrifuged for ten minutes at 1600 revolutions per minute. The sediment containing cells was used to undertake cytogenetic analysis. The supernatant, normally discarded, was frozen at −80 °C for further analysis. Se values were determined at the Medical Laboratory of the University Medical Centre Maribor. Electrothermal atomic absorption spectrometry (ETAAS) with graphite tube and Zeeman background correction using a Varian AA240Z Atomic Absorption Spectrometer (Varian Medical Systems, Inc., Palo Alto, California, USA) was performed. The analyzer was regularly calibrated and its accuracy checked using sample reagents as specified by the manufacturer. Se values were expressed in µg/L.

### 2.3. Statistical Analysis

Statistical analysis was performed using IBM SPSS Statistics for Mac, Version 23.0 (IBM Corp, Armonk, NY, USA). Descriptive statistics were employed to describe the main features of our data. When comparing groups of pregnant women, the χ^2^ test or Fisher’s exact test were used to test the frequency counts, the Student’s *t*-test to compare the means of continuous variables, and when the value distribution was not normal, the Mann–Whitney *U* test was used to test their median values. The Kolmogorov–Smirnov test was used for assessing data normality. A statistical power analysis was performed for sample size estimation. The projected sample size needed for power = 0.90 with this effect (Stata 13 for Mac) is approximately *n* = 206 patients. Thus, our proposed sample size was adequate for the testing of the main study objective. A multiple regression analysis was conducted to see if the confounding factors impact the outcomes of newborns. Statistical significance was set at *p* < 0.05.

This study was approved by the National Medical Ethics Committee of the Republic of Slovenia (Registration number: 87/11/11). All patients gave informed consent to participate in this study.

## 3. Results

**Figure 1:**
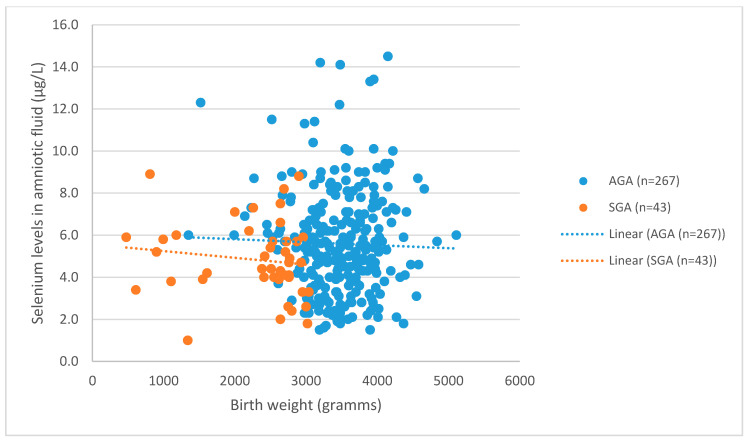
Distribution between Selenium levels and birth weight in small-for-gestational-age (SGA) and appropriate-for-gestational-age (AGA) newborns. A significant correlation was found between Se levels and SGA newborns (*t* = 2.080, *p* = 0.038).

327 samples of amniotic fluid were taken at 16/17 weeks of gestation. The number of normal live newborns was 310. In 17 (5.2%) cases, chromosomal abnormalities were found. Those patients were excluded from this study. The remaining patients were diagnosed and 267 (86.1%) newborns were assigned as appropriate for gestational age (AGA), and 43 (13.8%) newborns were diagnosed small for gestational age (SGA). Figure 1 depicts the distribution of birth weight related to Se levels.

We recorded the characteristics of mothers and newborns. These data include mother’s age, place of residence (city/countryside), parity, complications during pregnancy (pre-eclampsia, preterm delivery), and the sex of the child. Characteristics of pregnant women and newborns in the study are listed in Table 1. Women in our study were older (mean age 37.1 years in SGA and 35.9 years in AGA group). There were also more cases of pre-eclampsia (18.6% vs. 3.0%) in the SGA group than in the AGA group. Preterm deliveries were statistically significantly correlated with SGA newborns. There were (6.3% of pre-term AGA newborns and 27.9% pre-term SGA newborns. The gender of the newborns was also compared; in the SGA group, 28 girls (65.2%) and 15 boys (34.8%) (*p* = 0.011) were born.

### 3.1. Levels of Se in AGA and SGA

**Figure 2:**
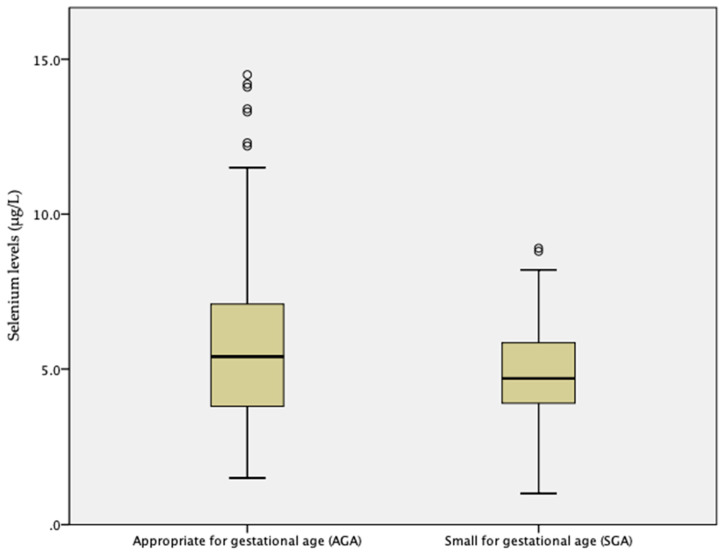
Comparison of mean Se concentrations in amniotic fluid. Se levels were significantly different between AGA and SGA newborns (*t* = −2.44; *p* = 0.017).

The mean and standard deviation of Se concentrations in amniotic fluid with AGA and SGA newborns were 5.6 ± 2.5 µg/L (min. value 1.5 µg/L; max. value 14.5 µg/L) in AGA and 4.8 ± 1.9 µg/L (min. value 1.0 µg/L; max. value 8.9 µg/L) in SGA, *t* = −2.44; *p* = 0.017. Figure 2 depicts a comparison of mean Se level values. There were significantly higher levels of pre-term births (*p* = 0.006, *t* = 2.98) in the SGA group, but this was not a statically significant correlation with Se levels in the sampled amniotic fluid (*p* = 0.099, *t* = 1.88).

Mean values of Se concentrations in cases with chromosomal abnormalities were 5.6 ± 2.3 µg/L; these cases were excluded from further analysis.

Values of Se in amniotic fluid slightly differed among pregnant women from town and from the surrounding area (5.7 ± 2.4 µg/L vs. 5.3 ± 2.5 µg/L; *p* > 0.05).

### 3.2. Pre-Eclampsia and Selenium Levels

**Figure 3:**
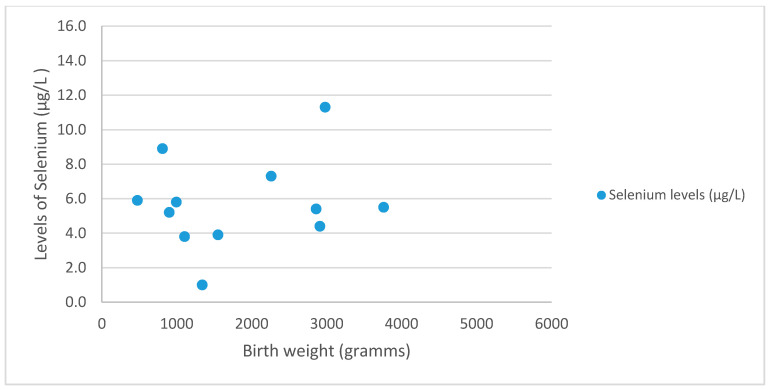
Distribution of birth weight in regard to Se levels in newborns with pre-eclampsia.

This dataset also analyzed the occurrence of pre-eclampsia and its connection to Se in SGA and AGA newborns. Pre-eclampsia was present in 3.0% newborns (*n* = 4) with AGA and in 18.6% (*n* = 8) newborns with SGA (*p* = 0.007, *t* = 2.83). However, Se levels were insufficient to predict statistically the occurrence of pre-eclampsia (*p* = 0.76, *t* = 0.309). Figure 3 depicts the distribution of Selenium levels in SGA and AGA newborns with pre-eclampsia. The mean levels of women with pre-eclampsia was 5.7 ± 2.6 µg/L.

Adjusting for different risk factors for SGA such as maternal age, nulliparity, and Zinc levels, it was shown that Selenium was the only significant factor impacting the outcome of a newborn (b = −0.152, s.e = 0.077; *p* < 0.048), the odds ratio of 0.859 indicates that with higher levels of Selenium found in amniotic fluid the odds of the newborn being small for gestational age are decreased.

## 4. Discussion

Our study demonstrated significantly lower Se concentration in the amniotic fluid in SGA fetuses. There were no differences between AGA and SGA newborns in regard to known risk factors such as age of the mother and nulliparity.

During pregnancy, maternal Se concentrations decrease (in the 1st trimester 65 µg/L; 3rd trimester 50 µg/L) [15]. Assessing trace elements could be a potential root of identifying women at risk of complications during pregnancy. Especially in early pregnancy, it is important to understand the impact of different factors contributing to complications in order to monitor the pregnancy appropriately. Wilson et al. [9] showed that in the serum plasma of women, lower levels of serum zinc, copper, and Se were protective and that plasma copper was the only factor to affect adverse pregnancy outcomes after adjusting statistically for levels of Se and Zinc [9]. This, however, is in contrast with findings described earlier linking Se to SGA newborns. Bizarea et al. showed [40] through their in vivo studies that there is an important antioxidant role for selenoproteins, which could contribute to the proper fetal development, leading to conclusions that on a molecular level there is evidence that SGA might be interconnected to Se levels. In vivo studies showed in a recent publication of Hofstee et al. that selenium deficiency did not impact in a mouse model the placental expression of antioxidants, but it did reduce fetal glucose concentrations leading to reduced fetal weight. This study showed that the reduced weight might be due to increased maternal thyroid hormone concentrations connected to low selenium levels and misfunctioning placental thyroid hormone, thus suggesting the mechanism of action in women with SGA babies is impaired thyroid metabolism and not placental antioxidant concentrations [19].

Previous research on Se concentrations and the development of SGA is inconsistent. A retrospective study reported low placental Se concentrations in 49 mothers affected by fetal growth restriction, compared to 36 healthy normal birth weight controls [41], whereas others reported higher [42,43] or unchanged concentrations [44]. Another retrospective study conducted on 81 SGA newborns also demonstrated infant plasma Se concentrations to be significantly lower compared to controls [45].

It has been suggested that adequate Se status is important for antioxidant defense and may be a potential factor in women at risk of pre-eclampsia [17]. Mistry et al. reported Se concentrations in pre-eclamptic pregnancies to be reduced in the mother’s serum [17,46,47] as well as in fetus amniotic fluid [13,48]. Exploring further, the important identified selenoproteins were 6 antioxidant glutathione peroxidases (GPx). The GPx activity in maternal and cord plasma as well as placental tissue was shown to be lower in pre-eclamptic pregnancies [15,30,46,49]. As previously described, amniotic fluid represents an important material for understanding the biological effects of Se [35,37]. Roy et al. reported Se levels in amniotic fluid samples. The levels were 6.8 ± 3.7 ng/mL in the control group and 6.7 ± 2.2 ng/mL in the pre-eclamptic group. The difference in the values of amniotic fluid Se between these two groups of patients, however, were not statistically significant [50]. In our study, Se levels in the amniotic fluid at 16/17 weeks in the SGA group of which 18.6% were pre-eclamptic patients were 4.8 ± 1.9 µg/L. The data on pre-eclampsia, however, are not fully conclusive. Cardoso de Silva [51] et al. showed that adjusting for other factors, such as age, ethnicity, education, parity, or smoking prevalence, serum levels between controls and women with gestational hypertension did not differ (*p* = 0.77). In 2016, a meta-analysis [52] also concluded that there is some evidence pointing towards the inverse association with selenium levels and the risk of pre-eclampsia. It was indicated that more prospective data is needed in order to support Se supplementation.

Our study found no statistically significant difference in regard to the levels of Se and the occurrence of pre-term birth. This finding is in concordance with a previously performed case-control study [53], but contrary to the findings of Yildiz et al. [36], showing that regardless of the tested material (mother blood, fetal blood, mother urine, amnion fluid), the levels of Se in late pregnancy were statistically significantly decreased in pre-term newborns. The molecular mechanisms of exposure to hyperoxia and inflammation which increases ROS could impact the mechanisms leading to premature birth. However, regardless of year of intensive research, the roles of Se, GPx, and selenoproteins are poorly understood in prematurity as well as oxidant injury [21]. In order to clearly be able to claim the role of trace elements in pre-term birth, roles of heavy metals should additionally be evaluated such as cadmium (Cd), which was found to be positively associated with the risk of early pre-term birth [54].

There are different routes of investigating levels of Se available. Many studies have focused on using highly accessible Se maternal plasma serum as a route for investigation. This is a possible route of investigation which yields many times comparable outcomes as amniotic fluid analysis [55]. Amniotic fluid analysis, however, was regarded to be especially important in heavy metal analysis and thus an understanding related directly to in-utero effects [56].

Interventional studies supplementing Se (using 1000 µg liquid *Se* per day) or placebo in patient with high risk for developing pre-eclampsia showed that the treatment group receiving Se had a decreased incidence of pregnancy-induced hypertension from 22.7% to 7.7% [57]. Moreover, Mesdaghinia et al. showed that Se supplementation (100 µg Se supplements as tablet between 17 and 27 weeks of gestation) in pregnant women at risk for IUGR resulted in an improved pulsatility index of <1.45 (*p* = 0.002) compared to those in the placebo group. In addition, changes in plasma levels of total antioxidant capacity (TAC) (*p* < 0.001), glutathione (GSH) (*p* = 0.008), and high-sensitivity C-reactive protein (hsCRP) (*p* = 0.004) in the Se group were significant compared to the placebo group [58]. Se supplementation in pregnant women was also shown to effectively reduce the incidence of premature membrane rupture [59].

Monitoring adolescents pregnant women nutrition and their trace element levels, Mistry et al. [60] showed that neither copper nor zinc differentiated between AGA and SGA newborns, but that low plasma selenium levels could contribute to placental antioxidant defense and thus contribute to SGA newborns [60]. There are wide differences in the Se intake across diverse populations, depending on the Se content of the soil and hence the Se content in staple foodstuffs. Se intake and food levels seem to be suboptimal in the northeastern part of Slovenia. Mičetić-Turk et al. [61] assessed serum Se level of mothers at birth to elucidate its impact on the Se content of umbilical cord serum of their newborns and of colostrum. There was a significant correlation between the Se content of maternal and umbilical serum, but no significant correlation was found between maternal serum Se and colostrum. The conclusion was that dietary Se intake for pregnant women in their population was borderline [61]. The levels of Se in amniotic fluid differed among pregnant women from town and from surrounding areas (5.7 ± 2.4 µg/L vs. 5.3 ± 2.5 µg/L; *t* = 1.360; *p* > 0.175), but the difference was not statistically significant. This may be explained by geographic variation. Indeed, some areas in Europe have already been identified as Se deficient areas, which might lead to more Se deficient pregnancies [22]. As there is a high incidence of SGA newborns in the northeastern area of Slovenia, further studies might also explore the role of Se dietary intake and environment impact on the presence of SGA newborns.

Our current understanding of different biochemical tests for placental function does not support the use of any of the standard methods of testing to be able to predict SGA newborns and placental dysfunction [62]. Therefore, recent findings are even more promising, showing, that trace elements might be a way forward to a predictive model for placental disfunction and growth restriction. Using trace elements as a prediction model for SGA and preterm birth seems to be a plausible future pathway of risk stratification. McKeating et al. [63] presented a model which enabled them to predict by the use of urine and plasma samples SGA and preterm birth. Within this study, 81.5% of the women identified then also had SGA newborns.

While further studies leading to more advanced understandings of the impact different trace elements have together on the risk for SGA newborns, preterm birth, pre-eclampsia, and gestational diabetes as well as their underlying causes such as placental dysfunction leading to growth restrictions.

Although our pilot study showed reliable initial data on Se levels, there are several limitations to this study. The first limitation is in regard to the number of cases assessed with pre-eclampsia. There was a small number of cases with pre-eclampsia in the study, and as such, any results on pre-eclampsia need to be validated in a larger cohort. An important limitation is based on not evaluating the effects of heavy metal levels on pregnancy outcomes, as these are interconnected to adverse outcomes in pregnancy [36]. Our study also did not adjust the levels of the trace element Se with potential seasonal changes, which was indicated to contribute to a variation of outcomes [63]. Further explorations regarding Se in connection to other trace elements and their impact on SGA development is needed as well as larger multifactorial analysis adjusting for risk factors leading to SGA. Our study also did not collect dietary data, which is emerging to might have an impact on SGA. As inflammation was linked to the development of SGA, recent studies focused on the influence of diet on the presence of SGA. A recent report [64] showed by longitudinally monitoring women during pregnancy that some eicosanoids and their fatty acid precursors were present in higher concentrations (41–97%) in SGA than in the control groups and that this could be linked to inflammation as a mechanisms of SGA newborns. Comparing other environmental factors on the development of SGA, a large study in Spain showed that the intake of vitamins D and B_3_ and ω-3 marine fatty acids as well as consumption of fruits, fish, and legumes can improve the impact of environmental factors leading to SGA [65]. Therefore, further studies assessing SGA should also evaluate dietary intake and its relation to trace elements and multidimensional effects on SGA newborns.

## 5. Conclusions

This study shows encouraging initial data to further explore Se levels in the amniotic fluid in patients referred to amniocentesis. It could provide a viable additional tool to develop risk stratification methods for women at risk for the development of SGA babies. Especially in regions with low levels of Se in their nutrition and a high incidence of SGA newborns, additional knowledge on the levels of Se in amniotic fluid could improve treatment and reduce the risk of low birth weight, the major cause of neonatal morbidity and mortality.

## Figures and Tables

**Table 1:** Obstetrical characteristics of pregnant women included in the study.

Characteristics	AGA (*n* = 267)	SGA (*n* = 43)	*p*-Value
Maternal age (year)	35.9 ± 4.2	37.1 ± 4.5	*p* = 0.11 (*t* = 1.59)
Nulliparity	24.3% (*n* = 65)	30.2% (*n* = 13)	*p* = 0.411 (*t* = 0.779)
Preterm birth	6.3% (*n* = 17)	27.9% (*n* = 12)	*p* = 0.006 (*t* = 1.65)
Boys Girls	55.4% (*n* = 148) 44.6% (*n* = 119)	34.8% (*n* = 15) 65.2% (*n* = 28)	*p* = 0.011 (*t* = 2.63)

## References

[1] Royal College of Obstetricians and Gynaecologists (2013). The Investigation and Manangement of the Small-for-Gestational-Age Fetus.

[2] Gordijn S.J., Beune I.M., Ganzevoort W. (2018). Building consensus and standards in fetal growth restriction studies. Best Pract. Res. Clin. Obstet. Gynaecol..

[3] Chiavaroli V., Castorani V., Guidone P., Derraik J.G.B., Liberati M., Chiarelli F., Mohn A. (2016). Incidence of infants born small- and large-for-gestational-age in an Italian cohort over a 20-year period and associated risk factors. Ital. J. Pediatr..

[4] Sharma D., Shastri S., Sharma P. (2016). Intrauterine Growth Restriction: Antenatal and Postnatal Aspects. Clin. Med. Insights Pediatr..

[5] Lewandowska M., Sajdak S., Lubiński J. (2019). The role of early pregnancy maternal selenium levels on the risk for small-for-gestational age newborns. Nutrients.

[6] Khalil A., Syngelaki A., Maiz N., Zinevich Y., Nicolaides K.H. (2013). Maternal age and adverse pregnancy outcome: A cohort study. Ultrasound Obstet. Gynecol..

[7] Kot K., Kosik-Bogacka D., Łanocha-Arendarczyk N., Malinowski W., Szymański S., Mularczyk M., Tomska N., Rotter I. (2019). Interactions between 14 elements in the human placenta, fetal membrane and umbilical cord. Int. J. Environ. Res. Public Health.

[8] Lewicka I., Kocyłowski R., Grzesiak M., Gaj Z., Oszukowski P., Suliburska J. (2017). Selected trace elements concentrations in pregnancy and their possible role—Literature review. Ginekol. Pol..

[9] Wilson R.L., Bianco-Miotto T., Leemaqz S.Y., Grzeskowiak L.E., Dekker G.A., Roberts C.T. (2018). Early pregnancy maternal trace mineral status and the association with adverse pregnancy outcome in a cohort of Australian women. J. Trace Elem. Med. Biol..

[10] Hofstee P., Cuffe J.S.M., Perkins A.V. (2020). Analysis of selenoprotein expression in response to dietary selenium deficiency during pregnancy indicates tissue specific differential expression in mothers and sex specific changes in the fetus and offspring. Int. J. Mol. Sci..

[11] Na J.Y., Seok J., Park S., Kim J.S., Kim G.J. (2018). Effects of selenium on the survival and invasion of trophoblasts. Clin. Exp. Reprod. Med..

[12] Liu P.J., Yao A., Ma L., Chen X.Y., Yu S.L., Liu Y., Hou Y.X. (2020). Associations of Serum Selenium Levels in the First Trimester of Pregnancy with the Risk of Gestational Diabetes Mellitus and Preterm Birth: A Preliminary Cohort Study. Biol. Trace Elem. Res..

[13] Mistry H.D., Broughton Pipkin F., Redman C.W.G., Poston L. (2012). Selenium in reproductive health. Am. J. Obstet. Gynecol..

[14] Bogden J.D., Kemp F.W., Chen X., Stagnaro-Green A., Stein T.P., Scholl T.O. (2006). Low-normal serum selenium early in human pregnancy predicts lower birth weight. Nutr. Res..

[15] Mihailovič M., Cvetkovč M., Ljubič A., Kosanovič M., Nedeljkovič S., Jovanovič I., Pešut O. (2000). Selenium and Malondialdehyde Content and Glutathione Peroxidase Activity in Maternal and Umbilical Cord Blood and Amniotic Fluid. Biol. Trace Elem. Res..

[16] Rayman M.P., Bode P., Redman C.W. (2003). Low selenium status is associated with the occurrence of the pregnancy disease preeclampsia in women from the United Kingdom. Am. J. Obstet. Gynecol..

[17] Mistry H.D., Wilson V., Ramsay M.M., Symonds M.E., Pipkin F.B. (2008). Reduced Selenium Concentrations and Glutathione Peroxidase Activity in Preeclamptic Pregnancies. Hypertension.

[18] Everson T.M., Kappil M., Hao K., Jackson B.P., Punshon T., Karagas M.R., Chen J., Marsit C.J. (2017). Maternal exposure to selenium and cadmium, fetal growth, and placental expression of steroidogenic and apoptotic genes. Environ. Res..

[19] Hofstee P., Bartho L.A., McKeating D.R., Radenkovic F., McEnroe G., Fisher J.J., Holland O.J., Vanderlelie J.J., Perkins A.V., Cuffe J.S.M. (2019). Maternal selenium deficiency during pregnancy in mice increases thyroid hormone concentrations, alters placental function and reduces fetal growth. J. Physiol..

[20] Richard K., Holland O., Landers K., Vanderlelie J.J., Hofstee P., Cuffe J.S.M., Perkins A.V. (2017). Review: Effects of maternal micronutrient supplementation on placental function. Placenta.

[21] Tindell R., Tipple T. (2018). Selenium: Implications for outcomes in extremely preterm infants. J. Perinatol..

[22] Zachara B.A. (2018). Selenium in Complicated Pregnancy. A Review. Adv. Clin. Chem..

[23] Hubel C.A. (1999). Oxidative stress in the pathogenesis of preeclampsia. Proc. Soc. Exp. Biol. Med..

[24] Šikić Pogačar M., Mičetić-Turk D. (2017). Vitamin D in Human Health. Acta Medico-Biotech..

[25] Fujimoto V.Y., Bloom M.S., Huddleston H.G., Shelley W.B., Ocque A.J., Browne R.W. (2011). Correlations of follicular fluid oxidative stress biomarkers and enzyme activities with embryo morphology parameters during in vitro fertilization. Fertil. Steril..

[26] Asemi Z., Jazayeri S., Najafi M., Samimi M., Mofid V., Shidfar F., Shakeri H., Esmaillzadeh A. (2012). Effect of Daily Consumption of Probiotic Yogurt on Oxidative Stress in Pregnant Women: A Randomized Controlled Clinical Trial. Ann. Nutr. Metab..

[27] Toblli J.E., Cao G., Oliveri L., Angerosa M. (2012). Effects of iron deficiency anemia and its treatment with iron polymaltose complex in pregnant rats, their fetuses and placentas: Oxidative stress markers and pregnancy outcome. Placenta.

[28] Yust-Katz S., Fisher-Shoval Y., Barhum Y., Ben-Zur T., Barzilay R., Lev N., Hod M., Melamed E., Offen D. (2012). Placental mesenchymal stromal cells induced into neurotrophic factor-producing cells protect neuronal cells from hypoxia and oxidative stress. Cytotherapy.

[29] Perkins A.V. (2011). Placental oxidative stress, selenium and preeclampsia. Pregnancy Hypertens..

[30] Zachara B.A., Dobrzynski W., Trafikowska U., Szymanski W. (2001). Blood selenium and glutathione peroxidases in miscarriage. BJOG.

[31] Punshon T., Li Z., Jackson B.P., Parks W.T., Romano M., Conway D., Baker E.R., Karagas M.R. (2019). Placental metal concentrations in relation to placental growth, efficiency and birth weight. Environ. Int..

[32] Ojeda M.L., Carreras O., Díaz-Castro J., Murillo M.L., Nogales F. (2019). High- and low- selenium diets affect endocrine energy balance during early programming. Toxicol. Appl. Pharmacol..

[33] Ojeda M.L., Nogales F., Membrilla A., Carreras O. (2019). Maternal selenium status is profoundly involved in metabolic fetal programming by modulating insulin resistance, oxidative balance and energy homeostasis. Eur. J. Nutr..

[34] Ojeda M.L., Nogales F., Serrano A., Murillo M.L., Carreras O. (2019). Maternal metabolic syndrome and selenium: Endocrine energy balance during early programming. Life Sci..

[35] Jalali L.M., Koski K.G. (2018). Amniotic fluid minerals, trace elements, and prenatal supplement use in humans emerge as determinants of fetal growth. J. Trace Elem. Med. Biol..

[36] Yıldırım E., Derici M.K., Demir E., Apaydın H., Koçak Ö., Kan Ö., Görkem Ü. (2019). Is the Concentration of Cadmium, Lead, Mercury, and Selenium Related to Preterm Birth?. Biol. Trace Elem. Res..

[37] Park J., Lee J.H., Yoon B.S., Jun E.K., Lee G., Kim I.Y., You S. (2018). Additive effect of bFGF and selenium on expansion and paracrine action of human amniotic fluid-derived mesenchymal stem cells 06 Biological Sciences 0601 Biochemistry and Cell Biology. Stem Cell Res. Ther..

[38] Verdenik I. (2000). Slovene reference standards for weight, length and head circumference at birth for given gestational age of population born in years 1987–96. Zdravniski Vestnik.

[39] Kramer M.S., McLean F.H., Olivier M., Willis D.M., Usher R.H. (1989). Body proportionality and head and length “sparing” in growth-retarded neonates: A critical reappraisal. Pediatrics.

[40] Bizerea T., Dezsi S., Marginean O., Stroescu R., Rogobete A., Bizerea-Spiridon O., Ilie C. (2018). The Link Between Selenium, Oxidative Stress and Pregnancy Induced Hypertensive Disorders. Clin. Lab..

[41] Klapec T., Ćavar S., Kasač Z., Ručević S., Popinjač A. (2008). Selenium in placenta predicts birth weight in normal but not intrauterine growth restriction pregnancy. J. Trace Elem. Med. Biol..

[42] Fall C.H.D., Yajnik C.S., Rao S., Davies A.A., Brown N., Farrant H.J.W. (2003). Micronutrients and Fetal Growth. J. Nutr..

[43] Zadrożna M., Gawlik M., Nowak B., Marcinek A., Mrowiec H., Walas S., Wietecha-Posłuszny R., Zagrodzki P. (2009). Antioxidants activities and concentration of selenium, zinc and copper in preterm and IUGR human placentas. J. Trace Elem. Med. Biol..

[44] Llanos M.N., Ronco A.M. (2009). Fetal growth restriction is related to placental levels of cadmium, lead and arsenic but not with antioxidant activities. Reprod. Toxicol..

[45] Strambi M., Longini M., Vezzosi P., Berni S., Buoni S. (2004). Selenium status, birth weight, and breast-feeding: Pattern in the first month. Biol. Trace Elem. Res..

[46] Atamer Y., Koçyigit Y., Yokus B., Atamer A., Erden A.C. (2005). Lipid peroxidation, antioxidant defense, status of trace metals and leptin levels in preeclampsia. Eur. J. Obstet. Gynecol. Reprod. Biol..

[47] Maleki A., Fard M.K., Zadeh D.H., Mamegani M.A., Abasaizadeh S., Mazloomzadeh S. (2011). The Relationship between Plasma Level of Se and Preeclampsia. Hypertens. Pregnancy.

[48] Dawson E.B., Evans D.R., Nosovitch J. (1999). Third-Trimester Amniotic Fluid Metal Levels Associated with Preeclampsia. Arch. Environ. Health An Int. J..

[49] Vanderlelie J., Venardos K., Clifton V.L., Gude N.M., Clarke F.M., Perkins A.V. (2005). Increased biological oxidation and reduced anti-oxidant enzyme activity in pre-eclamptic placentae. Placenta.

[50] Roy A.C., Ratnam S.S., Karunanithy R. (1989). Amniotic Fluid Selenium Status in Pre-Eclampsia. Gynecol. Obstet. Invest..

[51] Da Silva A.C., Martins-Costa S.H., Valério E.G., Lopes Ramos J.G. (2017). Comparison of serum selenium levels among hypertensive and normotensive pregnant women. Hypertens. Pregnancy.

[52] Xu M., Guo D., Gu H., Zhang L., Lv S. (2016). Selenium and Preeclampsia: A Systematic Review and Meta-analysis. Biol. Trace Elem. Res..

[53] Iranpour R., Zandian A., Mohammadizadeh M., Mohammadzadeh A., Balali-Mood M., Hajiheydari M. (2009). Comparison of maternal and umbilical cord blood selenium levels in term and preterm infants. Zhongguo Dang Dai Er Ke Za Zhi.

[54] Tsuji M., Shibata E., Morokuma S., Tanaka R., Senju A., Araki S., Sanefuji M., Koriyama C., Yamamoto M., Ishihara Y. (2018). The association between whole blood concentrations of heavy metals in pregnant women and premature births: The Japan Environment and Children’s Study (JECS). Environ. Res..

[55] Lewicka I., Kocyłowski R., Grzesiak M., Gaj Z., Sajnóg A., Barałkiewicz D., von Kaisenberg C., Suliburska J. (2019). Relationship between pre-pregnancy body mass index and mineral concentrations in serum and amniotic fluid in pregnant women during labor. J. Trace Elem. Med. Biol..

[56] Caserta D., Mantovani A., Ciardo F., Fazi A., Baldi M., Sessa M.T., la Rocca C., Ronchi A., Moscarini M., Minoia C. (2011). Heavy metals in human amniotic fluid: A pilot study. Prenat. Diagn..

[57] Han L., Zhou S.M. (1994). Selenium supplement in the prevention of pregnancy induced hypertension. Chin. Med. J..

[58] Mesdaghinia E., Rahavi A., Bahmani F., Sharifi N., Asemi Z. (2017). Clinical and Metabolic Response to Selenium Supplementation in Pregnant Women at Risk for Intrauterine Growth Restriction: Randomized, Double-Blind, Placebo-Controlled Trial. Biol. Trace Elem. Res..

[59] Tara F., Rayman M.P., Boskabadi H., Ghayour-Mobarhan M., Sahebkar A., Yazarlu O., Ouladan S., Tavallaie S., Azimi-Nezhad M., Shakeri M.T. (2010). Selenium supplementation and premature (pre-labour) rupture of membranes: A randomised double-blind placebo-controlled trial. J. Obstet. Gynaecol..

[60] Mistry H.D., Kurlak L.O., Young S.D., Briley A.L., Broughton Pipkin F., Baker P.N., Poston L. (2014). Maternal selenium, copper and zinc concentrations in pregnancy associated with small-for-gestational-age infants. Matern. Child Nutr..

[61] Mičetić-Turk D., Rossipal E., Krachler M., Li F. (2000). Maternal selenium status in Slovenia and its impact on the selenium concentration of umbilical cord serum and colostrum. Eur. J. Clin. Nutr..

[62] Heazell A.E., Whitworth M., Duley L., Thornton J.G. (2015). Use of biochemical tests of placental function for improving pregnancy outcome. Cochrane Database Syst. Rev..

[63] McKeating D.R., Clifton V.L., Hurst C.P., Fisher J.J., Bennett W.W., Perkins A.V. (2020). Elemental Metabolomics for Prediction of Term Gestational Outcomes Utilising 18-Week Maternal Plasma and Urine Samples. Biol. Trace Elem. Res..

[64] Welch B.M., Keil A.P., van ’t Erve T.J., Deterding L.J., Williams J.G., Lih F.B., Cantonwine D.E., McElrath T.F., Ferguson K.K. (2020). Longitudinal profiles of plasma eicosanoids during pregnancy and size for gestational age at delivery: A nested case-control study. PLoS Med..

[65] Martínez-Galiano J.M., Amezcua-Prieto C., Cano-Ibañez N., Olmedo-Requena R., Jiménez-Moleón J.J., Bueno-Cavanillas A., Delgado-Rodríguez M. (2020). Diet as a counteracting agent of the effect of some well-known risk factors for small for gestational age. Nutrition.

